# Comparative Analysis of Intestinal Morphology and Gut Microbiota of *Spinibarbus sinensis* Under Different Aquaculture Systems

**DOI:** 10.3390/biology13110869

**Published:** 2024-10-25

**Authors:** Qi Deng, Zhifeng Feng, Jin Xiang, Hao Wu, Xin Yang, Zhou Zhang, Cheng Li, Xiaofei Cheng, Min Xie, Shaoming Li

**Affiliations:** Hunan Fisheries Science Institute, Changsha 410153, China; dengqi22222@gmail.com (Q.D.); fengzhifeng@sina.com (Z.F.); xiangjin1023@163.com (J.X.); wh17380133463@163.com (H.W.); yangxinhzau@163.com (X.Y.); zz19961022@hotmail.com (Z.Z.); licheng1969001@sina.com (C.L.); chengxiaofei19@126.com (X.C.)

**Keywords:** *Spinibarbus sinensis*, aquaculture systems, intestinal morphology, 16S rRNA, gut microbiota

## Abstract

Fish gut health is influenced by various factors, with the environment being a significant one. *S. sinensis* is a key aquaculture species in China, yet research on the impact of different aquaculture systems on its intestinal health remains limited. This study aims to explore the changes in intestinal morphology and gut microbiota of *S. sinensis* under two aquaculture systems. The juveniles of *S. sinensis* were divided into two groups and cultured in traditional ponds (CT) and an in-pond tank culture system (JY), with equal amounts of feed provided daily over a 72-day experimental period. The results showed no significant differences in growth performance metrics, including the specific growth rate, weight gain rate, hepatosomatic index, and viscerosomatic index between the two groups. In terms of intestinal morphology, the JY group villus width was significantly wider than the CT group, and the number of goblet cells in the CT group was significantly higher than that of the JY group (*p* < 0.05), which suggested that the fish in the JY group may have better intestinal nutrient absorption capacity, while the water quality in the CT group may be worse. The 16S rRNA gene sequencing analysis of the gut microbiota showed that the JY group had a significantly higher Shannon index compared to the CT group (*p* < 0.05), indicating greater species richness and evenness. Principal Coordinates Analysis (PCoA) revealed a distinct clustering of gut microbiota between the two groups. At the phylum level, the relative abundance of Fusobacteriota was significantly higher in the CT group, whereas Bacteroidota and Proteobacteria were significantly higher in the JY group (*p* < 0.05). Furthermore, KEGG pathway predictions indicated differences in the potential metabolic capabilities of the gut microbiota between the two groups (*p* < 0.05). Overall, this study is the first to conduct a comparative analysis of the growth performance, intestinal tissue morphology, and gut microbiota of *S. sinensis* under two different aquaculture systems, which has valuable implications for the further optimization of aquaculture practices.

## Simple Summary

This study investigated the changes in the intestinal morphology and gut microbiota of *Spinibarbus sinensis* under two aquaculture systems: traditional pond and in-pond tank culture systems. The results demonstrated significant differences in villus width and goblet cell numbers between the two groups regarding intestinal morphology. Additionally, there were significant differences in gut microbiota richness, diversity, and predicted potential functions. The specific mechanisms by which an aquaculture system influences the intestine of *S. sinensis* merit further exploration.

## 1. Introduction

Pond aquaculture is the primary method of freshwater aquaculture in China, covering an area of 2,624,878 hectares, which constitutes 52% of the total freshwater aquaculture area. With the continuous expansion of aquacultural scale, traditional pond farming faces severe challenges such as high water consumption, the deterioration of aquaculture water quality, and pollution from aquaculture wastewater, which severely constrain its sustainable development [1]. To address these challenges, China has recently developed various new pond aquaculture systems, such as the in-pond tank culture system [2] and the land-based container aquaculture system [3]. The in-pond tank culture system consists of a culture tank, a capture and aeration module, a waste collection module, and a vertical flow constructed wetland, and the effective rearing area takes up about 10.05% of the pond area. The land-based container aquaculture system mainly consists of aquaculture containers with a length, width, and height of approximately 6.1 m, 2.4 m, and 2.9 m, respectively; at the bottom of the containers is a 10° inclined plane (and it is therefore easy to collect pollutants). However, most studies on these aquaculture systems are still in the exploratory stage.

The primary function of fish intestines is the digestion and absorption of nutrients, and the internal surface area of the intestines is a key morphological factor affecting absorption [4]. Studies have shown that some factors can affect intestinal morphology, such as the environment and nutrition [5,6]. Moreover, fish intestines contain a large number of microorganisms that play crucial roles in host growth, immunity, and energy metabolism [7,8,9]. In fish, intestinal microbiota are influenced by a variety of factors, including host factors, microbial factors, and other environmental factors [10]. Recent research has shown that environmental factors affect the intestinal microbiota of fish. For instance, the richness and diversity of intestinal microbiota of juvenile cobia (*Rachycentron Canadum*) increases and then decreases under hypoxic stress [11]. Nile tilapia (*Oreochromis Niloticus*) from aquaculture centers exhibit higher intestinal microbiota diversity than wild Nile tilapia from Lake Tana [12]. Similarly, Ussuri whitefish (*Coregonus ussuriensis*) exposed to 10 °C and 25 °C temperatures possessed a lower *Lactobacillus* abundance compared to exposure to a temperature of 19 °C [13]. The culture system significantly impacted the gut microbiota of bighead carp (*Hypophthalmichthys nobilis*), resulting in differences in community structure, abundance, and potential metabolic functions, and altered the host’s gut metabolism, especially in pathways related to amino acid metabolism [14]. Therefore, it is necessary to study the impact of different environments on fish gut health, which is of great significance for the sustainable development of fish pond aquaculture.

*S. sinensis* is an important cultured fish species in China and one of the most abundant fish species in the upper and middle Yangtze River water body. Its meat is tender and rich in proteins, fats, carbohydrates, vitamins, and minerals such as calcium, phosphorus, and iron [15,16,17]. Current research on *S. sinensis* mainly focuses on growth performance, respiration function, and vulnerability to angling [18,19,20]. There are currently few published articles on the intestinal tract of *S. sinensis* [21]. This study aims to investigate the differences in intestinal morphology and gut microbiota of *S. sinensis* under traditional pond and in-pond tank culture systems, and preliminarily discuss the potential causes of these differences, in order to provide a reference for the further optimization of different aquaculture systems.

## 2. Materials and Methods

### 2.1. Fish Management

In this study, *S. sinensis* were collected and cultured at the aquaculture base of the Hunan Fisheries Science Institute (Changsha, China), which is located at 28°17′0.46″ N, 113°1′4.35″ E. The aquaculture systems were divided into two groups: a traditional pond group and an in-pond tank culture system group; three replicates were set up in each group and the same groundwater source was used. Each aquaculture system was stocked with 6000 *S. sinensis* juveniles (2000 per replicate), with the stocking density referring to the study of Lu et al. [2]. The traditional pond has an area of 667 m^2^, an average water depth of 1.5 m, is surrounded by a cement slope protection with a mud structure as the substrate, and a stocking density of approximately 3 fish/m^3^ (0.39 kg/m^3^). The in-pond tank culture system consists of a culture tank, a capture and aeration module, a waste collection module, and a vertical flow constructed wetland, with a cylindrical container (diameter 4.0 m, height 1.9 m) as the main culture area, an effective culture water volume of about 20 m^3^, and a stocking density of approximately 100 fish/m^3^ (13.02 kg/m^3^). The experimental period was from 26 June 2023 to 6 September 2023; the entire experimental period from the beginning to the end is the growth period of the juveniles. During the experiment, the daily feeding amount was 3% of the fishes’ body weight, with extruded feed being provided twice a day (8:00 AM and 5:00 PM). The feed was procured from Aonong company (Xiamen, China), and its composition was crude protein ≥ 40%, crude fat ≥ 5%, crude fiber ≤ 8%, crude ash ≤ 15%, total phosphorus ≥ 1.2%, lysine ≥ 2.2%, and moisture ≤ 10%. The average initial weight of the fish was 130.19 ± 12.35 g, and the average body length was 18.5 ± 1.2 cm. The water temperature in the two systems during the breeding period ranged from 28 to 35 °C and the pH value was between 7.3 and 7.8, which is suitable for fish survival.

### 2.2. Sample Collection

At the end of the rearing experiment, the fish were fasted for 24 h. The traditional pond group was designated as the CT group, and the in-pond tank culture system group as the JY group. Nine fish were randomly selected from each replicate culture system, with 27 fish in each group euthanized with MS-222 (100 mg/L, Sigma-Aldrich, St. Louis, MO, USA) and dissected on ice. The body length, weight, and viscera weight of the fish were measured for growth performance analysis. Nine fish of a similar weight were selected from each group for histological analysis (three fish per replicate), with distal intestine samples preserved in 4% paraformaldehyde fixative. Six fish were randomly selected from each group for intestinal microbiota analysis (two fish per replicate), and the intestinal contents of each fish were collected into a 2 mL sterile centrifuge tube, snap-frozen in liquid nitrogen for 30 min, and then transferred to a −80 °C freezer. The fish that were used for growth performance analysis, histological analysis, and intestinal microbiota analysis were sampled independently.

### 2.3. Growth Performance Analysis

Growth performance parameters include the specific growth rate (SGR), the weight gain rate (WGR), the hepatosomatic index (HSI), and the viscerosomatic index (VSI). The formulas for these calculations are as follows:

SGR (%) = [(*ln*W_t_ − *ln*W_0_)/d] × 100

WGR (%) = (W_t_ − W_0_)/W_0_ × 100

HSI (%) = W_h_/W_t_ × 100

VSI (%) = W_v_/W_t_ × 100

In the formulas, W_t_ represents the final weight of the fish, W_0_ represents the initial weight of the fish, W_h_ represents the weight of the liver, and W_v_ represents the weight of the viscera. All weights are measured in grams (g), and d represents the number of days the fish were reared.

### 2.4. Preparation and Observation of Intestinal Tissue Sections

Intestinal tissue samples were fixed in paraformaldehyde fixative for 24 h, dehydrated in an alcohol gradient, cleared with xylene, and embedded in paraffin. The samples were then sectioned into 5 μm thick slices and stained with hematoxylin-eosin (HE). One slice was used at a similar position in the intestine for each fish, and the images were observed and imaged using an Eclipse Ci-L microscope (Nikon, Tokyo, Japan). The images were analyzed using Image-Pro Plus 6.0 software (Media Cybernetics Corporation, Rockville, MD, USA). All areas in each image were used for observation and counting. The villus height (VH), villus width (VW), and muscle layer thickness (MT) were measured in μm and averaged. The number of goblet cells was the total number in each image.

### 2.5. Intestinal Microbes Sequencing

Genomic DNA from the samples was extracted using the MagPure Soil DNA LQ Kit (MAGBIO Genomics, Germantown, MD, USA) according to the manufacturer’s instructions. The concentration and purity of the DNA were measured using a NanoDrop 2000 (Thermo Fisher Scientific, Waltham, MA, USA) and agarose gel electrophoresis, and the extracted DNA was stored at −20 °C. The bacterial 16S rRNA gene was amplified by PCR using the extracted genomic DNA as a template, with specific primers and Takara Ex Taq high-fidelity enzyme. Universal primers 343F (5′-TACGGRAGGCAGCAG-3′) and 798R (5′-AGGGTATCTAATCCT-3′) [22] were used to amplify the V3-V4 variable regions of the 16S rRNA gene for bacterial diversity analysis. The PCR amplification conditions were based on the method of Zhang et al. [9]. The PCR products were verified by agarose gel electrophoresis and purified using AMPure XP beads (Beckman Coulter, Brea, CA, USA). The purified product was used as a template for a second-round PCR, followed by another purification with magnetic beads. The purified second-round products were quantified using a Qubit, adjusted in concentration, and sequenced. Sequencing was performed on the Illumina NovaSeq 6000 platform to generate 250 bp paired-end reads by OE Biotech Co., Ltd. (Shanghai, China).

### 2.6. Bioinformatic Analysis

The raw sequencing data were obtained in FASTQ format. The raw sequences were first trimmed to remove primer sequences using Cutadapt (https://gitcode.com/gh_mirrors/cu/cutadapt/overview, accessed on 23 October 2024). Quality filtering, denoising, merging, and chimera removal were performed using DADA2 (https://github.com/benjjneb/dada2, accessed on 23 October 2024) [23] within the QIIME 2 (2020.11) [24] pipeline, generating representative sequences and ASV (Amplicon Sequence Variant) abundance tables. Representative sequences were classified using the Silva (version 138) database. Species annotation was performed using the q2-feature-classifier plugin with default parameters. Venn diagrams and histograms of the abundance of the intestinal bacterial communities and the rarefaction curves of samples were constructed using R language tools. Alpha diversity analysis was performed using QIIME 2, including the Shannon index, chao1 index, observed_species index, and Simpson index. Beta diversity was calculated using weighted and unweighted UniFrac distances [25], and principal coordinate analysis (PCoA) was performed. Differential analysis was performed using *t*-test statistics based on the R package. LDA effect size (LEfSe) and biomarkers were used to analyze the differential abundance of bacterial groups at the genus level. The functional prediction of the gene sequences was performed using PICRUSt2 (2.3.0b0) based on the KEGG (Kyoto Encyclopedia of Genes and Genomes) database to analyze functional abundance and differences.

### 2.7. Statistical Analysis

All experimental data are presented as mean ± standard deviation (mean ± SD). After the confirmation of normal distribution and homogeneity of variance, inter-group data were analyzed using independent sample *t*-test statistics with SPSS 20.0 (Statistical Product Service Solutions) software, with *p* < 0.05 considered statistically significant.

## 3. Results

### 3.1. Growth Performance

**Figure 1:**
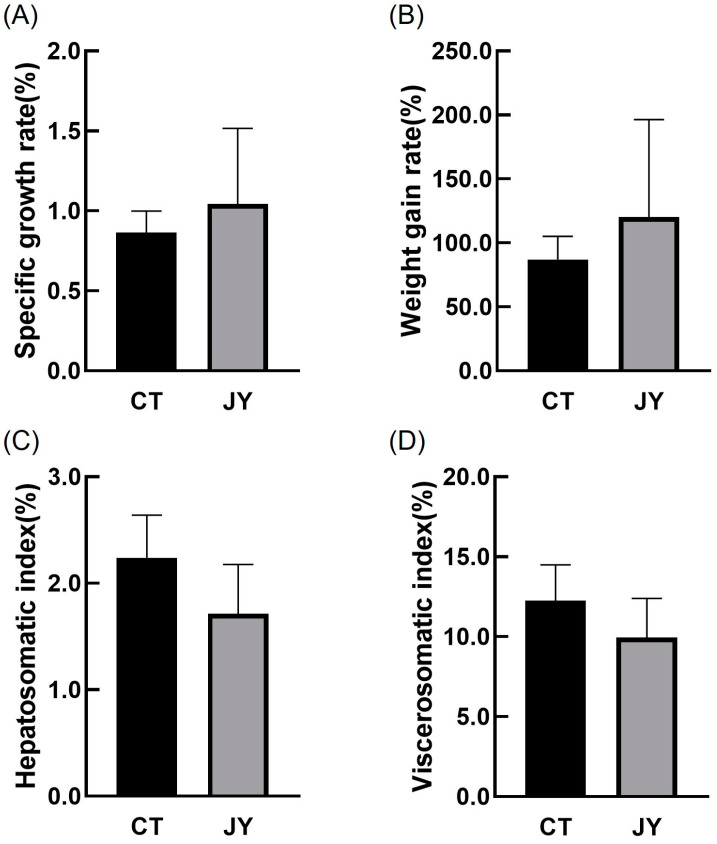
The growth performance of *S. sinensis* under different aquaculture systems. (**A**–**D**) in the figure represent the specific growth rate (SGR), weight gain rate (WGR), hepatosomatic index (HSI), and viscerosomatic index (VSI), respectively. The data are presented as mean ± SD (n = 27).

The growth performance data of *S. sinensis* under different aquaculture systems are presented in Figure 1. The results indicate that there were no significant differences in specific growth rate, weight gain rate, hepatosomatic index, or viscerosomatic index between the CT group and the JY group (*p* > 0.05).

### 3.2. Intestinal Tissue Morphology

**Figure 2:**
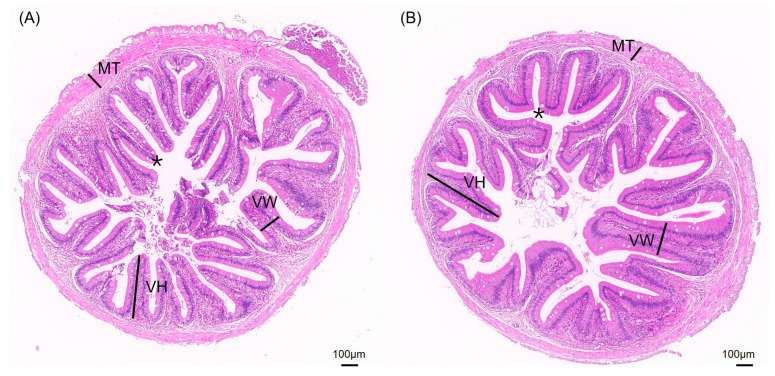
Intestinal tissue sections of *S. sinensis* under different aquaculture systems. (**A**,**B**) are HE slices of intestines from groups CT and JW, respectively. VH, VW, MT, and asterisks (*) in the figure represent villus height, villus width, muscular thickness, and goblet cells, respectively.

**Figure 3:**
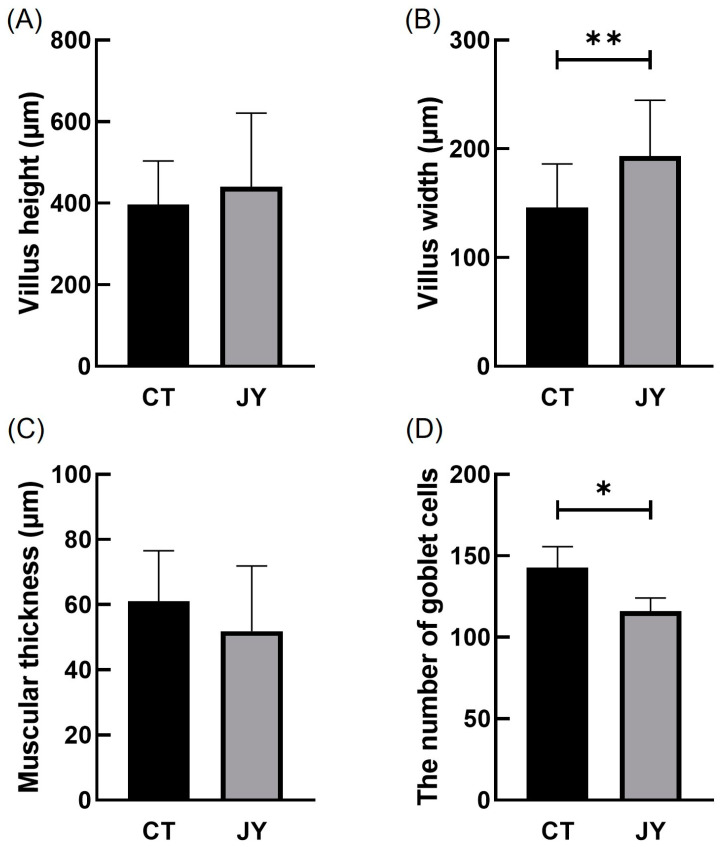
Villus height (**A**), villus width (**B**), muscular thickness (**C**), and the number of goblet cells (**D**) of *S. sinensis* intestinal tissue under different aquaculture systems. The asterisks (*) and double asterisks (**) indicate significant differences (*p* < 0.05) and an extremely significant difference (*p* < 0.01) between different groups.

The impact of different aquaculture systems on the intestinal tissue morphology is shown in Figure 2. The intestinal tissue sections of *S. sinensis* from both groups reveal intact and clear villus structures, with neatly arranged epithelial cells and complete cellular structures. To better investigate intestinal health, we measured villus height (VH), villus width (VW), muscle layer thickness (MT), and the number of goblet cells (Figure 3). The results show that the villus width (VW) in the JY group was extremely significantly higher than in the CT group (*p* < 0.01) and the number of goblet cells in the CT group was significantly higher than in the JY group (*p* < 0.05), while there were no significant differences in villus height (VH) and muscle layer thickness (MT) between the two groups (*p* > 0.05).

### 3.3. Intestinal Microbes

#### 3.3.1. Intestinal Microbial ASVs

**Figure 4:**
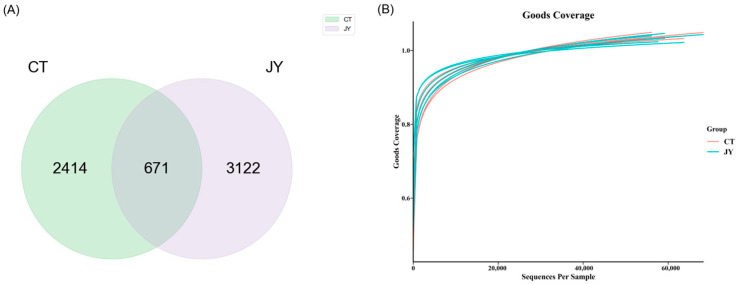
Intestinal microbial ASVs of *S. sinensis* under different aquaculture systems: (**A**) Venn diagram showing the number of shared and unique ASVs in CT, and JY groups. (**B**) The Goods Coverage rarefaction curves of all samples; the abscissa is the sequencing depth and the ordinate is the exponential value.

The genomic DNA was extracted from the intestinal content samples in each sterile centrifuge tube, and the DNA was amplified and purified before 16S sequencing. A total of 739,354 reads were obtained from 12 samples in the two groups (6 replicates in each group), and the number of reads for each sample ranged from 56,972 to 69,330. The sequences were clustered into ASVs, and a Venn diagram (Figure 4A) was constructed to analyze the ASVs of the two groups. As shown in Figure 4A, there were 671 shared ASVs between the CT and JY groups, with 2414 unique ASVs in the CT group and 3122 unique ASVs in the JY group. A total of 26 phyla, 57 classes, 142 orders, 232 families, and 407 genera were identified. The Goods Coverage rarefaction curves (Figure 4B) indicated that the high-throughput sequencing depth covered the majority of species in the samples, and was therefore suitable for further analysis.

#### 3.3.2. Intestinal Microbial Diversity of *S. sinensis* Under Different Aquaculture Systems

**Figure 5:**
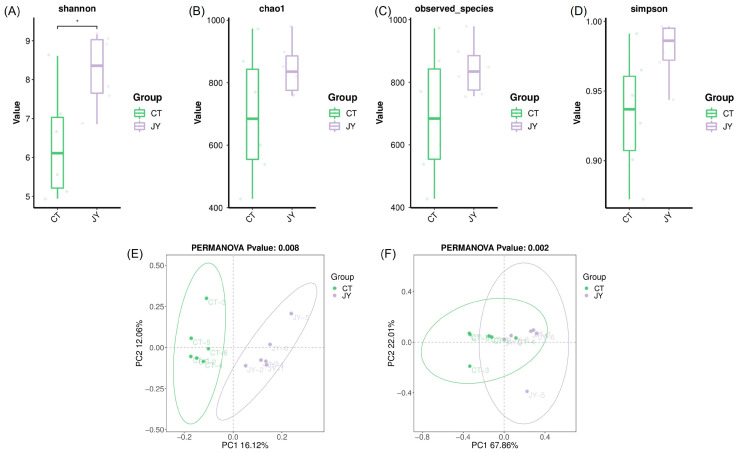
Intestinal microbial diversity of *S. sinensis* under different aquaculture systems: (**A**–**D**) in the figure represent the Shannon index, chao1 index, observed species, and Simpson index for alpha diversity analysis, (**E**,**F**) in the figure represent the principal coordinate analysis (PCoA) based on unweighted and weighted Unifrac distance algorithms for beta diversity analysis. The asterisks (*) indicate significant differences (*p* < 0.05) between the CT group and the JY group.

The alpha diversity of the intestinal microbiota was assessed using the Shannon index (Figure 5A), chao1 index (Figure 5B), observed species index (Figure 5C), and Simpson index (Figure 5D). The results showed that the mean values of all indices in the JY group were higher than those in the CT group, with the Shannon index of the CT group being significantly lower than that of the JY group (*p* < 0.05). However, there were no significant differences in the chao1 index, observed species index, or Simpson index (*p* > 0.05). Additionally, principal coordinate analysis (PCoA) based on unweighted Unifrac (Figure 5E) and weighted Unifrac (Figure 5F) distance algorithms was used to reflect the beta diversity of the samples. The results demonstrated a clear distinction between the CT and JY groups, with significant differences (*p* < 0.05).

#### 3.3.3. Species Composition and Abundance of Intestinal Microbes

**Figure 6:**
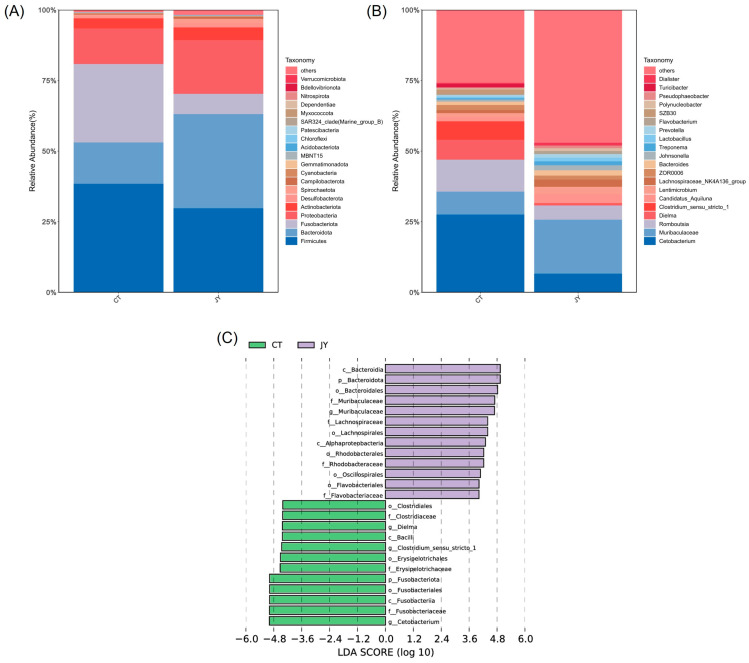
Species composition and abundance of intestinal microbes of *S. sinensis* under different aquaculture systems. Relative abundances of dominant microbial phyla (**A**) and genera (**B**) in the intestine of the CT group and JY group. (**C**) Linear discriminant analysis effect size (LEfSe) score in the intestinal microbiota community of the two groups from Lefse-PICRUSt.

At the phylum level, the dominant phyla in both the CT and JY groups were Firmicutes, Fusobacteriota, Bacteroidota, and Proteobacteria, with the combined relative abundance of these four phyla exceeding 90% (Figure 6A). The results indicated significant differences in the relative abundances of Fusobacteriota (27.6% vs. 6.8%), Bacteroidota (14.8% vs. 33.6%), and Proteobacteria (12.7% vs. 19.2%) between the CT and JY groups (*p* < 0.05), while there was no significant difference in Firmicutes (38.2% vs. 29.4%) (*p* > 0.05). At the genus level, the main dominant genera in the CT and JY groups were *Cetobacterium*, *Romboutsia*, *Muribaculaceae*, and *Dielma*, with the combined relative abundance of these four genera exceeding 30% (Figure 6B). It should be noted that *Muribaculaceae* is a family, but the sequencing results are shown as an uncultured genus in the database, so *Muribaculaceae* is used instead of the genus name in this study. The results showed significant differences in the relative abundances of *Cetobacterium* (27.4% vs. 6.3%), *Muribaculaceae* (8.2% vs. 19.2%), and *Dielma* (6.9% vs. 0.5%) (*p* < 0.05), while there was no significant difference in *Romboutsia* (11.2% vs. 4.8%). LEfSe analysis also revealed that *Cetobacterium* was significantly enriched in the CT group, while *Muribaculaceae* was significantly enriched in the JY group (Figure 6C).

#### 3.3.4. Predicted Functions of Intestinal Microbes

**Figure 7:**
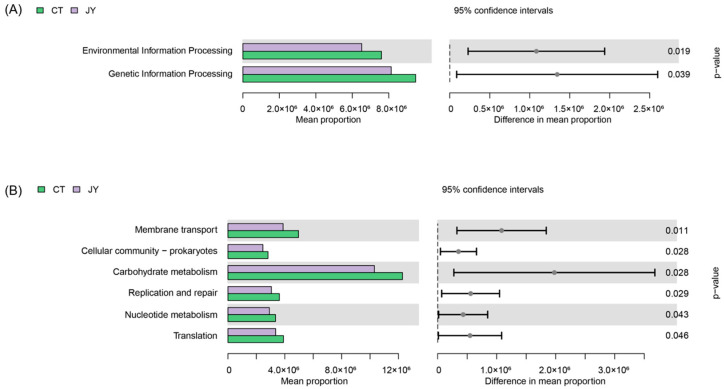
Predicted functions of intestinal microbes of *S. sinensis* under different aquaculture systems. (**A**) Relative abundances of predicted genes in the metagenome of KEGG pathways level 1, (**B**) relative abundances of predicted genes in the metagenome of KEGG pathways level 2.

The functional annotation of the intestinal microbiota genes using the KEGG database revealed that at Level 1 (Figure 7A), the intestinal microbiota genes of both groups were associated with six metabolic pathways. There were no significant differences in the average abundance of genes related to metabolism, cellular processes, human diseases, and organismal systems between the two groups (*p* > 0.05), whereas there were significant differences in genetic information processing and environmental information processing (*p* < 0.05). At Level 2 (Figure 7B), significant differences were observed in the intestinal microbiota of the two groups, with the most enriched two pathways being carbohydrate metabolism and membrane transport (*p* < 0.05).

## 4. Discussion

Indicators such as the Specific Growth Rate (SGR), the Weight Gain Rate (WGR), the Hepatosomatic Index (HSI), and the Viscerosomatic Index (VSI) provide a direct reflection of fish growth performance [14]. The experimental results showed no significant differences in these four indicators between the CT and JY groups, indicating that there is no significant difference in the growth performance of *S. sinensis* under the two aquaculture systems. This finding is consistent with previous research on carp growth performance under similar conditions [3]. Furthermore, despite the JY group having a much higher stocking density of 100 fish/m^3^ compared to 3 fish/m^3^ in the CT group, the growth rates of both groups were nearly identical. We hypothesize that the relatively short rearing period may have contributed to the lack of significant differences in growth rates at these stocking densities, as juvenile fish were in a growth stage throughout the experimental period; moreover, this suggests that the stocking density used in our study is relatively reasonable.

The intestine plays a crucial role in nutrient digestion and absorption in aquatic animals. Changes in intestinal tissue structure are essential for the optimal utilization of dietary nutrients [26]. The differences in the intestinal morphology of *S. sinensis* under two aquaculture systems are mainly reflected in the width of villus (VW) and the number of goblet cells, while there were no significant differences in the villus height (VH) and muscular thickness (MT) (*p* > 0.05). Interestingly, the villus width (VW) in the JY group was significantly greater than in the CT group (*p* < 0.05). A wider villus width suggests that the JY group’s intestines may have a better nutrient absorption capacity, because the wider villus width increases the surface area of the villus, expanding the area for absorbing nutrients, a phenomenon also observed in controlled container culture of snakehead fish [27]. The intestinal mucosa’s epithelium is composed of a single layer of columnar epithelial cells, interspersed with numerous goblet cells that secrete mucus to protect the organ both mechanically and biologically [28]. Traditional ponds are prone to water quality deterioration. For example, studies have shown that the lack of appropriate nitrification and denitrification bacterial strains in ponds hinders the normal reproduction of beneficial algae and affects the aquatic environment of the largemouth bass [29]. The histological results showed that the number of goblet cells in the CT group was significantly higher than in the JY group (*p* < 0.05), possibly due to the water environment of traditional pond culture, which prompts the mucosal epithelium to produce more goblet cells to ensure intestinal protection; however, the specific impact mechanism needs further exploration.

In order to further compare and analyze the intestinal tract of *S. sinensis* under different aquaculture systems, we focused on changes in the gut microbiota. Previous studies have shown that factors such as diet composition [30,31], environmental factors [32,33], different growth stages [34], and host selection [35] can influence the structure and quantity of gut microbiota, thereby affecting the organism’s nutrient metabolism, immune regulation, and growth development. Alpha and beta diversity indices are generally used to assess fish intestinal microbiota diversity [36]. In this study, the Shannon index for alpha diversity was significantly higher in the JY group compared to the CT group, indicating higher species richness and an evenness of intestinal microbiota in the in-pond tank culture system. This finding is similar to studies on the gut microbiota of Indian major carp [37] and bighead carp [38], which showed different gut microbiota diversity under different culture systems. Generally, the richness and diversity of the intestinal flora of asymptomatic fish are higher than those of diseased fish [39,40]. Therefore, although there was no significant difference in growth performance, we speculated that the fish in the CT group might be in a poorer health state than the fish in the JY group due to the different water environment, which is consistent with our analysis of the intestinal tissue results. Principal Coordinate Analysis (PCoA) results also showed significant differences in beta diversity between the two groups, indicating the distinct clustering of gut microbiota under different aquaculture systems; considering that the two groups were from the same batch of fish and fed the same commercial feed, the factor causing this significant difference between habitats may be system differences, which is similar to the results recently found in bighead carp [14]. The above results showed that the gut microbial diversity of *S. sinensis* varied significantly under the two aquaculture systems; this may be related to the water environment under different systems, but further verification is still needed. For example, this hypothesis could be tested by simulating similar water conditions of CT in JY in the future.

The term core microbiota describes microbes that are consistently present in a particular habitat [41]. At the phylum level, the core microbiota in the intestines of *S. sinensis* were Firmicutes, Fusobacteriota, Bacteroidota, and Proteobacteria, similar to findings in various fish species [42,43]. The relative abundance results showed no change in the core microbiota species under both aquaculture systems, though there were differences in their proportions at the phylum level. Firmicutes was the dominant phylum in both groups, likely because it is one of the main phyla in freshwater fish intestines [42]. Fusobacteriota and Bacteroidota showed significant differences between the groups, with Bacteroidota being significantly higher in the JY group. An increased abundance of Bacteroidota has been linked to improved fermentation and nutrient absorption [44] and is positively correlated with plant-based diets [45]. Therefore, we hypothesized that the higher abundance of Bacteroidetes in the JY group may be related to better intestinal nutrient absorption, which is interestingly similar to our intestinal tissue studies. Conversely, the amount of Fusobacteriota was significantly higher in the CT group. Fusobacteria have been widely reported in fish; for example, Fusobacteria can promote purine metabolism in the host intestine [46] and tea polyphenols can increase the abundance of Fusobacteria in the intestine of spotted bass under fish oil oxidative stress [47]. We speculate that the environment of traditional ponds may be conducive to the proliferation of Fusobacteria in the fish intestine, although the exact mechanism needs further verification. At the genus level, significant differences were observed in *Cetobacterium* and *Muribaculaceae* between the CT and JY groups. *Cetobacterium* was significantly more abundant in the CT group and is known to produce vitamin B_12_, enhancing the stability of the intestinal microbiota network and improving resistance to pathogen infection [48]. This suggests that the gut microbiota of the CT group may offer better protection against pathogen infection. Conversely, *Muribaculaceae* was significantly more abundant in the JY group. *Muribaculaceae* is beneficial, with studies showing its reduction in response to new shellfish toxins damaging the intestines of mice [49], and its increase with metformin treatment improving inflammation and liver injury in rats [50]. Thus, the gut microbiota of the JY group may offer better anti-inflammatory capabilities. In short, the significant difference in the abundance of intestinal bacteria in the above two systems may be the result of environmental selection by intestinal bacteria, but the specific influencing factors require further verification.

At Level 1, the predicted KEGG pathway results showed significant differences in the Genetic Information Processing and Environmental Information Processing pathways between the two groups, likely related to the abundance differences in Fusobacteriota and Bacteroidota. This finding is similar to reports on the intestinal microbiota of Malaysian catfish [51]. Level 2 includes 44 categories such as cell growth and death, transcription, and development. Carbohydrate metabolism is the pathway category with significant differences and the highest enrichment in the two groups, indicating that the metabolic functions of the intestinal microbiota differ between the fish in the two systems. We speculate that this difference may be mainly related to the genus Bacteroides. It has been reported that the carbohydrate metabolism pathway is related to the enrichment of the genus Bacteroides, which in turn affects the nutrition and health of the host [52].

## 5. Conclusions

This study is the first to analyze the growth performance, intestinal tissue morphology, and gut microbiota of *S. sinensis* under two different aquaculture systems. Our findings revealed significant differences in intestinal morphology, microbial community structure, abundance ratios, and predicted potential metabolic functions between the two systems. However, it should be noted that our current research aims to reveal the differences in *S. sinensis* under two aquaculture systems. The specific influencing factors are still difficult to determine because there are too many influencing factors under different aquaculture systems, and further research is needed in the future. In summary, the results of this study provide a preliminary basis for understanding the intestinal morphology, microbial composition, and diversity of *S. sinensis* under different aquaculture systems. These findings have valuable implications for the further optimization of aquaculture practices, such as further studying the mechanism of action of specific microorganisms in growth and health and identifying potential bacteria that can be used as probiotics to improve the growth performance and disease resistance of *S. sinensis*.

## Data Availability

The raw read sequences obtained from sequencing were deposited in the Sequence Read Archive (SRA) under BioProject accession number PRJNA1137139 (SUB14598253) and will be released after 12 August 2025. The datasets generated and analyzed during the current study are available from the corresponding author upon reasonable request.
